# Design and Analyses of a MEMS Based Resonant Magnetometer

**DOI:** 10.3390/s90906951

**Published:** 2009-09-02

**Authors:** Dahai Ren, Lingqi Wu, Meizhi Yan, Mingyang Cui, Zheng You, Muzhi Hu

**Affiliations:** State Key Laboratory of Precision Measurement Technology and Instruments, Department of Precision Instruments and Mechanics, Tsinghua University, Beijing, 100084, China; E-Mails: yanmz04@gmail.com (M.Z.Y.); Cmy9964@gmail.com (M.Y.C.); yz-dpi@mail.tsinghua.edu.cn (Z.Y.); hu-mz@mails.tsinghua.edu.cn (M.H.)

**Keywords:** magnetometer, MEMS, capacitance, Lorentz force, simulation

## Abstract

A novel design of a MEMS torsional resonant magnetometer based on Lorentz force is presented and fabricated. The magnetometer consists of a silicon resonator, torsional beam, excitation coil, capacitance plates and glass substrate. Working in a resonant condition, the sensor’s vibration amplitude is converted into the sensing capacitance change, which reflects the outside magnetic flux-density. Based on the simulation, the key structure parameters are optimized and the air damping effect is estimated. The test results of the prototype are in accordance with the simulation results of the designed model. The resolution of the magnetometer can reach 30 nT. The test results indicate its sensitivity of more than 400 mV/μT when operating in a 10 Pa vacuum environment.

## 1. Introduction

Magnetometers are sensors for magnetic field detection, which are often employed in industrial, oceanographic and biomedical fields. They are key sensors in geomagnetic field measurement, magnetic pattern imaging, mineral deposit detection, etc. [1]. In some biomedical applications, with the high requirements in sensitivity and accuracy, magnetometers should also be small enough and have low power consumption, whereas the performances of most present sensors are not satisfactory. MEMS technology provides an opportunity to solve this problem.

Currently, the most popular principles in MEMS magnetometers are the Hall Effect, magneto-resistance and the fluxgate effect [2]. However, Hall Effect magnetometers have low sensitivity and large temperature shifts; the sensors based on magnetoresistance are only appropriate to measure intense magnetic fields and fluxgate effect magnetometers are very difficult to fabricate. In this paper, a novel MEMS torsional resonant magnetometer based on the Lorentz Force principle is put forward. Such a sensor does not require magnetic materials, which makes it easier to fabricate by MEMS process. The magnetometer does not need an additional Au electrode on its surface and that the multi-turn excitation coil increase its driving torque. Compared with the MEMS surface resonant magnetometer developed by the Qinetiq Ltd. [3], our torsional structure has a much larger Quality Factor and excitation torque, which can magnify the vibration amplitude and facilitate the output signal detection. In the case of the Micromechanical Resonant Magnetic Sensor made by the University of California at Los Angeles, the piezoresistive sensing method had been employed for the detection of the vibration amplitude [4]. Compared with the capacitive displacement detection used in our design, such a method is sensitive to temperature, which limits its application range. In addition, compared with the magnetometer realized by Delft University of Technology [5], our fabrication process is quite different. Also the new structures such as low-resistivity silicon resonator and multi-turn excitation coil are put forward, by which the sensor’s performance is greatly improved.

## 2. Operation Principle

**Figure 1.**
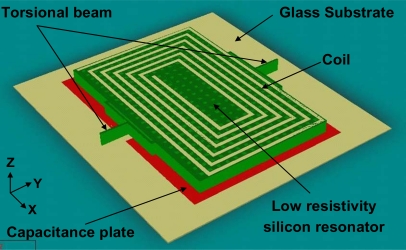
Structure of the MEMS torsional resonant magnetometer.

A low-resistivity silicon structure is suspended over the glass substrate by torsional beams, as indicated in Figure 1. Above the silicon structure, a 1 μm thick, 50 μm wide multi-turn excitation coil is deposited. The free ends of the torsional beams are fixed by anchors bonded onto the glass substrate. The Au capacitance plates are fabricated directly onto the glass substrate to form the capacitor with the low-resistivity silicon resonator, of which the resistivity is between 0.001 Ω·cm and 0.004 Ω·cm.

**Figure 2.**
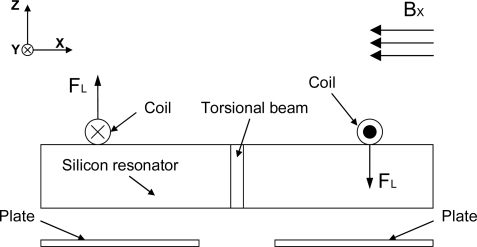
Operation principle of the MEMS torsional resonant magnetometer.

The operation principle is illustrated in Figure 2. If a DC current *I* is introduced into the excitation coil and a magnetic flux-density *B_x_* in x-direction is presented, for one turn of the excitation coil with length *L_c_* in y-direction, the Lorentz forces perpendicular to the silicon plane are:

(1) $$F_{L}=I\cdot L_{c}\cdot B_{x}$$

The Lorentz forces are generated on both sides of the resonator with opposite directions. Therefore the torque caused by the Lorentz forces is of the same direction, which twists the torsional beams.

If a sinusoidal current shown in (2) passes through the excitation coil instead of a DC current, the silicon resonator will vibrate around the torsional beams due to the alternating directions of the Lorentz force *F_L_* in (3):

(2) $$i=I_{0}\;\text{sin}\;2\pi \;\mathrm{ft}$$

(3) $$F_{L}=i\cdot L_{c}\cdot B_{x}=I_{0}L_{c}B_{x}\;\text{sin}\;2\pi \;\mathrm{ft}$$

When the frequency *f* of the current is equal to the resonant frequency of the silicon resonator, the vibration amplitude will dramatically increase due to the resonance and the high quality factor of the structure. Then this vibration amplitude will be converted into the differential change of the two sensing capacitances as shown indicated in Figure 2. Here, a capacitance detection circuit could be used to measure the capacitance change that reflects the value of the magnetic flux-density [3].

For the Lorentz Force Magnetometer, the input is the magnetic flux-density *B_x_* in x-direction and the output is the capacitance change Δ*C*. The transfer function can be expressed as in (4):

(4) $$\Delta C=S_{M}\;S_{\varphi }\;S_{C}\;B_{x}=\frac{\partial M}{\partial B}\cdot \frac{\partial \varphi }{\partial M}\cdot \frac{\partial (\Delta C)}{\partial \varphi }\cdot B_{x}$$

Here, *S_M_*, *S_φ_* and *S_C_* respectively stand for the transfer function from the magnetic flux-density *B* to the excitation torque *M*, from the excitation torque *M* to the torsional angle *φ*, and from the torsional angle *φ* to the capacitance change Δ*C*. When the structural parameters of the resonant magnetometer are determined, it can be proved that *S_M_*, *S_φ_* and *S_C_* are approximately constant under small deflection condition [6]. Therefore, the capacitance change Δ*C* has a linear relationship with the magnetic flux-density *B_x_*. The output signal of the capacitance detection circuit can accurately represent the magnetic flux-density *B_x_* in x-direction when the torsional angle of the resonator is in an appropriate range. For instance, when the resonator surface area *S* is 3,000 μm × 2,000 μm and the capacitance plate distance *d_0_* is 15 μm, the linearity of the sensor can be within 1.0% when the rotation angle does not exceed 6 × 10^−4^ rad.

## 3. Fabrication Process

**Figure 3.**
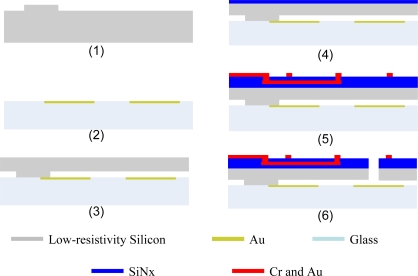
Fabrication process of the MEMS torsional resonant magnetometer.

The fabrication process of the prototype is based on the anodic bonding of low-resistivity silicon and a Pyrex glass substrate. The movable structures are released by the BOSCH process using ICP (Inductive Coupled Plasma) instead of a sacrificial layer process, which greatly increases the production yield. In addition, the low-resistivity silicon resonator directly acts as the electrode of the sensing capacitances. Therefore the magnetometer does not need additional electrode plates on the silicon resonator, which largely simplifies the fabrication process without influencing the performance. The fabrication process is described as indicated in Figure 3.

1. A step with the height of 10 μm is etched on the backside of the low-resistivity silicon to generate the capacitance plate distance.
2. The 0.3 μm thick Au capacitance plates are fabricated on the glass substrate by lift off process.
3. After the anodic bonding of the low-resistivity silicon and the Pyrex glass substrate, the silicon wafer is grinded from 200 μm to 70 μm thickness by the CMP (Chemical Mechanical Planarization) process.
4. A 1 μm layer of SiN_x_ is deposited on the surface of low-resistivity silicon for insulation.
5. One μm layers of Cr and Au are deposited and patterned to fabricate the multi-turn coil.
6. The movable structures are released by BOSCH etching process using ICP.

**Figure 4.**
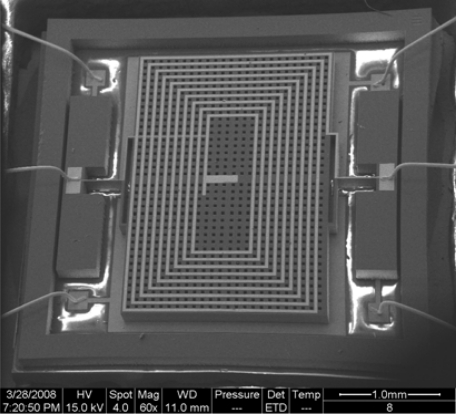
SEM photo of the fabricated MEMS torsional resonant magnetometer.

A SEM photograph of the fabricated magnetometer is shown in Figure 4.

## 4. Design and Simulation

**Figure 5.**
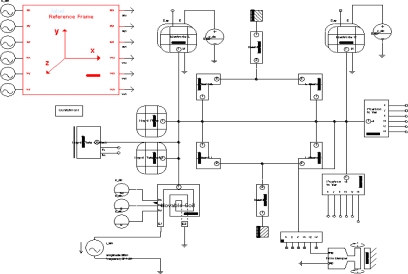
Simulation structure of the magnetometer with CoventorWare.

The simulation of the MEMS torsional resonant magnetometer is performed by the ARCHITECT SaberSketch editor in CoventorWare, in which the electrical, electro-mechanical, mechanical and magnetic parts build the magnetometer structure together, as indicated in Figure 5. Using the ARCHITECT SaberSketch, we can directly simulate the magnetometer’s performance and observe the output signals when doing a parametric study. This simulation process avoids the FEM meshing method, which largely increases the simulation speed.

**Figure 6.**
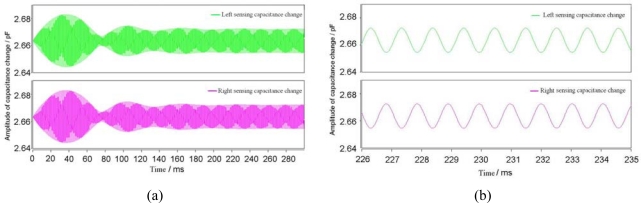
Transient change of the two sensing capacitances.

In this paper, the simulation aims to demonstrate the working principle, examine its sensitive direction, optimize the structure dimensions and estimate the influence of the damping effect of the air gap between the resonator and the capacitance plate. To demonstrate the operation principle of the magnetometer and the validity of the simulation model, a 30 mA amplitude AC current is introduced into the excitation coil. When the horizontal magnetic flux-density to be measured is 50 μT, the simulation results of the two sensing capacitances are illustrated in Figure 6.

Figure 6(a) shows the two sensing capacitances at 300 ms from the time when the horizontal magnetic flux-density is applied. The amplitude of the sensing capacitance change Δ*C* becomes stable after about 150 ms. The transition time and overshoot shown in Figure 6(a) are related to the system damping ratio and the damping from environment, such as the air. Figure 6(b) shows the left and right sensing capacitances by magnifying the time interval. The curves of the two sensing capacitances have the same amplitude but reverse phase, which demonstrates the differential capacitance change.

**Figure 7.**
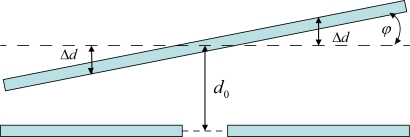
Differential change of the two sensing capacitances.

When designing the structure of the MEMS torsional resonant magnetometer, some key parameters that largely influence the device performance should be first determined. Most of these parameters are set up by resolution and accuracy requirements or fabrication and power consumption restrictions. The differential change of the two sensing capacitances is illustrated in Figure 7. Formula (5) shows the capacitance change in the case of small deflection [6], where *ε* denotes dielectric constant, *S* is the resonator surface area, *L* is the resonator length, *W* is the resonator width, *d_0_* is the capacitance plate distance and Δ*d* is the average resonator displacement in z-direction.

(5) $$\Delta C=\frac{\varepsilon S}{2\left(d_{0}-\Delta d\right)}-\frac{\varepsilon S}{2\left(d_{0}-\Delta d\right)}=\frac{\varepsilon S\;\Delta d}{d_{0}^{2}-{\left(\Delta d\right)}^{2}}\underset{\text{small deflections}}{\approx }\frac{\varepsilon \mathrm{LW}\Delta d}{d_{0}^{2}}$$

According to (5), *S* should be enlarged and *d_0_* should be decreased to get high amplitude of Δ*C*. However, since the low-resistivity silicon needs to be anodic bonded with the Pyrex glass, the high voltage applied during this process may generate breakdown of the air between the low-resistivity silicon and the capacitance plates. In addition, the CMP process that is used to decrease the thickness of the resonator may crush the silicon wafer because of the pressure added on the surface. Due to such restrictions, we set the minimum *d_0_* to 10 μm and the maximum *S* to 6 mm^2^. To reach the maximum excitation torque, The *W/L* value needs to be in the range of *1 < W/L < 2* [5].

### 4.1. Resonant Frequency

**Figure 8.**
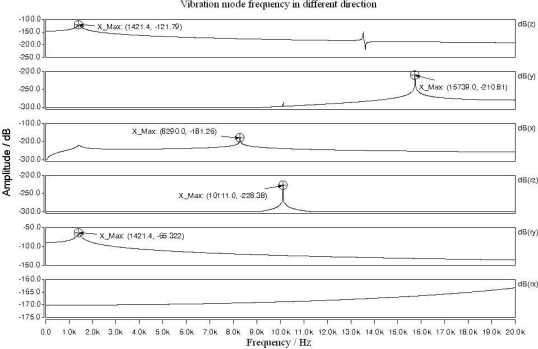
Different vibration modes of the silicon resonator of the magnetometer.

By examining the first-order resonant frequencies and the intervals between the resonant frequencies in different vibration modes, the sensitive direction of the sensor can be determined. According to the magnetometer’s working principle, the first-order vibration mode of the silicon resonator should be in ry-direction (rotating the y-axis). When the torsional beam length is 400 μm, beam width 20 μm and silicon thickness 60 μm, the simulated vibration modes of the silicon resonator are illustrated in Figure 8. When the silicon resonator is vibrating in ry-direction, its ends will have displacement in both ry-direction and z-direction. Therefore the first-order resonant frequency in the two directions have the same value of 1421.4 Hz as shown in Figure 8. The resonant frequencies in x-, y- and rz-directions are higher than 8 kHz, which are far from the ry-direction resonant frequency and will not influence the operation vibration mode. The simulation result proves that the designed magnetometer is only sensitive to the magnetic flux-density *B_x_* in the horizontal x-direction, which makes the torsional structure vibrating in ry-direction.

The theoretical resonant frequency in ry-direction can be obtained from (6):

(6) $$f\approx \sqrt{24\;\frac{{\mathrm{Gw}}^{3}}{\rho_{\mathrm{Si}}{\mathrm{LW}}^{3}l}\;\left(\frac{1}{3}-\frac{64}{\pi^{5}}\;\frac{w}{h}\sum_{n=1,3,5,\infty }^{\infty }\left(\text{tanh}\left(\frac{n\pi h}{2w}\right)/n^{5}\right)\right)}/2\pi$$

Herein *ρ_Si_* = 2300 kg/m^3^ denotes the low-resistivity silicon density, *G* = 6.9 × 10^10^ Pa is the silicon shear modulus, *w* is the beam width and *l* is the beam length. Based on the parameters above, the calculated resonant frequency is 1,333.7 Hz. The tested resonant frequency of the fabricated magnetometer with the same dimensions is around 1,320 Hz, which is in accordance with the simulation and theoretical data.

### 4.2. Structure Parameters

This simulation investigates the influence of the resonator thickness to the resonant frequency and the output capacitance change Δ*C*, which helps to optimize the structure dimensions. The elastic coefficient of the torsional beam can be written as:

(7) $$k=\frac{{\mathrm{Ghw}}^{3}}{l}\;\left(\frac{1}{3}-\frac{64}{\pi^{5}}\;\frac{w}{h}\sum_{n=1,3,5,\infty }^{\infty }\text{tanh}\frac{n\pi h}{2w}/n^{5}\right)$$

**Figure 9.**
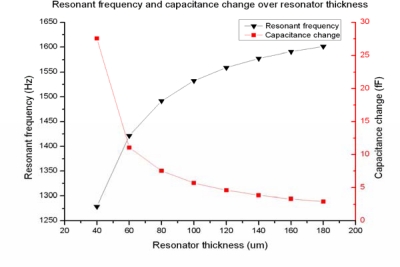
Resonant frequency and Δ*C* output over resonator thickness.

When the resonator thickness is reduced, the elastic coefficient decreases and the beams are easier to twist, which would undoubtedly generate a higher Δ*C*. Figure 9 shows the resonant frequency and Δ*C* over different resonator thickness, applying a 50 μT magnetic flux-density and 30 mA amplitude AC current. When the silicon resonator is thinned, the resonant frequency decreases but the Δ*C* output increases, both of which have a nonlinear relationship with the resonator thickness. In addition, not only does thin resonator increase the amplitude of Δ*C* as illustrated in Figure 9, but also it simplifies the fabrication process, especially in BOSCH etching. This will largely increase the fabrication quality of our MEMS based magnetometer.

**Figure 10.**
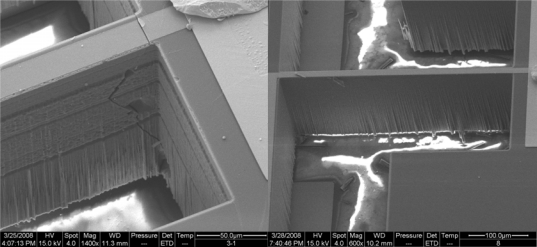
Lateral quality of the beam for a 180 μm thick resonator.

Figure 10 shows the lateral quality of the fabricated magnetometer when the resonator is around 180 μm thick. The jagged lateral surface of the torsional beams will significantly influence the mechanical properties. Therefore thin resonator would not challenge the BOSCH etching process and thus guarantee the good mechanical properties. However, thin resonator will decrease the resonant frequency as indicated in Figure 9, which will generate more noise in low frequency. In addition, the silicon wafer is also easy to break during the CMP process. In view of these opposite impacts of the resonator thickness, the 60 μm thickness is chosen as the proper dimension.

**Figure 11.**
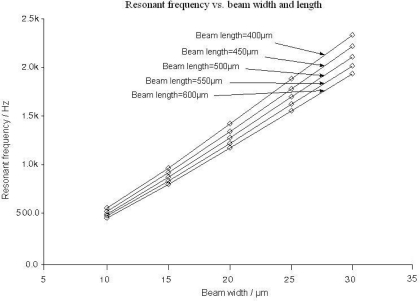
Resonant frequency over beam width and length.

According to (7), in order to decrease the elastic coefficient *k* and improve the sensitivity, long and narrow torsional beams are required. However, this requirement will also decrease the resonant frequency, as shown in Figure 11. Considering the noise in low frequency, it is wise to select the value of *l* and *w* with the resonant frequency higher than 1 kHz.

For the fabricated sensor with the beam length *l* = 500 μm, the beam width *w* = 20 μm and the resonator thickness = 60 μm, the tested resonant frequency is around 1,320 Hz, which agrees with the simulation results in Figure 11. In addition, the performance test results by the capacitance detection circuit, which will be mentioned later, indicate that the amplitude of the output capacitance change is enough to reach a high sensitivity with the structural parameters mentioned above.

### 4.3. Squeeze Film Damping Effect

**Figure 12.**
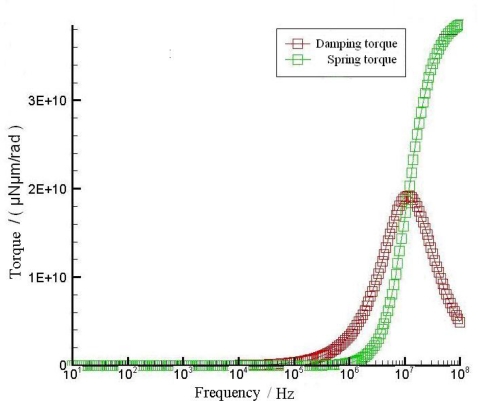
Frequency characteristics of the damping torque and spring torque.

We have estimated the influence of the squeeze film damping effect and investigated the necessity for the sensor to work in a vacuum environment. The thickness of the air layer between the resonator and substrate is in the order of tens of microns and the area is around 3,000 μm × 2,000 μm. Such an air gap will exert a torque on the plate. This torque has components both in-phase and out-of-phase with the velocity of the resonator. The velocity in-phase component is the damping torque, and the out-of-phase component is the spring torque. Both terms are frequency-dependent and deduced from appropriate physical models of fluids in the regions near the resonator [7–8]. The frequency characteristics of the damping torque and spring torque are illustrated in Figure 12, which is achieved by the CoventorWare.

As Figure 12 indicates, the spring torque rises rapidly as the frequency increases. The air captured in the cavity between the resonator and glass substrate is squeezed. Because there is no real closed cavity in this case, at low frequencies air can escape with little resistance and the torque is small. At high frequencies the air is held captive by its own inertia. Essentially, there is not enough time for the air to move out of the way as the resonator oscillates. Therefore the air compresses, resulting in a spring torque. Since the damping torque itself is caused by viscous stresses, if the gas compresses and does not move much, the damping torque will be lower. This explains why the damping torque gets smaller as the frequency increases. According to the above analyses, the squeeze film damping effect of such a thin, large-area air layer would decrease the vibration amplitude, especially in high frequency. To reduce this negative effect, some damping holes are etched through the silicon resonator, which allow the escape of the air captured between the resonator and the glass substrate. These damping holes are essential even if they will lower the value of initial capacitance which may decrease the sensor’s resolution.

**Figure 13.**
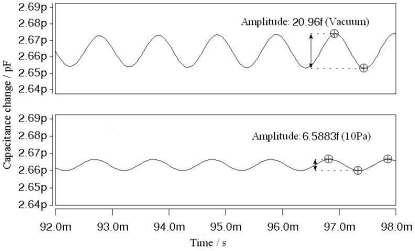
Δ*C* output in a 10 Pa pressure and vacuum environment.

To further reduce the squeeze film damping effect of the air layer, the sensor should be packaged in an approximate vacuum environment. Figure 13 shows the simulation results of the Δ*C* output in a 10 Pa pressure environment comparing with that in pure vacuum. The Δ*C* output of the approximately vacuum packaged sensor is 6.5883 fF, which is 31% of the Δ*C* output in pure vacuum environment without squeeze film damping effect. When the magnetometer operates in a normal environment pressure, the Δ*C* output would decrease more significantly. Therefore it is essential for the magnetometer to work in a vacuum environment.

### 4.4. Driving and Detection Circuit

**Figure 14.**
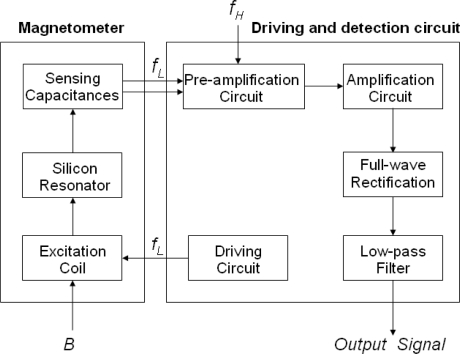
The schematic block diagram of the driving and detection circuit.

The schematic diagram of the driving and detection circuit of the magnetometer is illustrated in Figure 14. The sinusoidal signal *f_L_* generated by the driving circuit is applied into the excitation coil. The *f_L_* has the same frequency as the structure’s resonant frequency, which resonates the magnetometer and periodically changes the sensing capacitances. The high-precision and low-noise pre-amplification circuit is employed to convert the weak signal of the sensing capacitance to small voltage signal. In this process, the high-frequency carrier *f_H_* is applied to suppress the low-frequency noise. By the follow-up amplification, full-wave rectification and low-pass filter circuit, the AC small voltage is converted into DC voltage, whose amplitude reflects the magnetic flux-density to be measured.

## 5. Test and Analyses

### 5.1. The Initial Static Capacitance

In order to investigate the quality of the fabrication process, the value of the initial static capacitance needs to be measured and compared with the simulation results. The value of the initial static capacitance is related to the capacitance plate distance *d_0_*, the resonator length *L* and the resonator width *W*. The CoventorWare DC Operating Point Analysis is used to simulate the value of the initial static capacitance, taking into account the damping holes and the edge fringing field approximation. The result indicates the value of the initial static capacitance is 2.663 pF, as indicated in Figure 6. After the fabrication of the MEMS torsional resonant magnetometers, the initial capacitances of 5 different types (M1∼M5) of magnetometers were tested. The results are shown in Table 1. All of the fabricated magnetometers had the value of initial static capacitance between 2.6 pF and 2.8 pF, which was in great accordance with the simulation result.

### 5.2. The Squeeze Film Damping Effect

**Figure 15.**
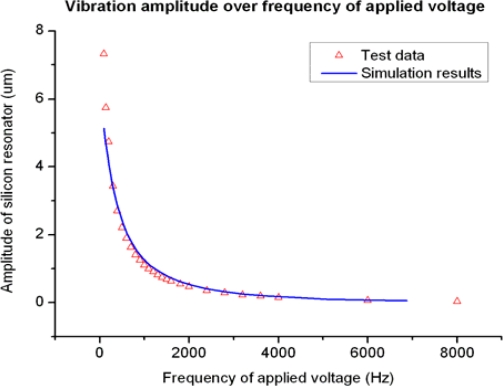
Vibration amplitude of the magnetometer over voltage frequency.

According to the previous discussion on the squeeze film damping effect, the structure of damping holes on the silicon resonator is essential to the magnetometer. Figure 15 shows the simulation and test results of the vibration amplitude without damping holes. During the test on the fabricated sensor with no damping holes, an AC voltage of different frequencies is applied between the resonator and one of the capacitance plates. The alternating electrostatic force generated by the applied voltage excites the vibration and the amplitude is tested by the Laser Vibrometer. With the same setup, we also simulated the performance of the magnetometer without damping holes. As shown in Figure 15, the test result is in good agreement with the simulation data. No resonant peak appeared as the frequency swept from 100 Hz to 8 kHz, mainly due to the squeeze film damping effect generated by the air gap. This result means the high quality factor of the resonator was not fully utilized and the resolution of the sensor could be quite low due to the lack of damping holes.

**Figure 16.**
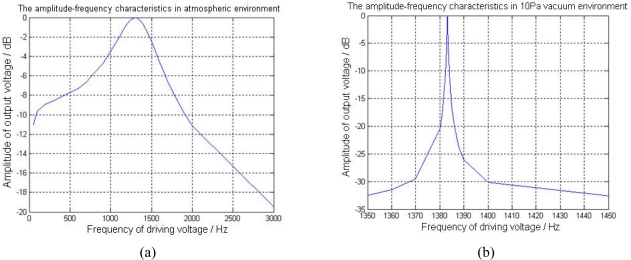
The amplitude-frequency characteristics in atmospheric and 10 Pa vacuum environment.

To further increase the magnetometer’s sensitivity, the sensor needs to work in the vacuum environment. For the magnetometer with the structure of damping holes, we tested the prototype’s amplitude-frequency characteristics as in Figure 16. By applying a 30 μT horizontal magnetic flux-density using 3-axis Helmholtz coil, we tested the output voltage when sweeping the driving voltage frequency. The y-axis in Figure 16 shows the normalized amplitude divided by the maximum output voltage. For the sensor operating in a 10 Pa vacuum environment as in Figure 16(b), the tested Quality Factor Q is around 2,530.1, which is nearly 1,000 times of the Q value 2.62 measured in normal pressure as shown in Figure 16(a). The test results certified the great importance of the vacuum operating environment.

When the magnetic flux-density *B_x_* is alternating with frequency *f_B_*, the Lorentz force *F_L_* has the frequency components of *f + f_B_* and *f − f_B_* according to (3), which means that the driving force *F_L_* has a frequency shift from the resonant frequency and thus decrease the amplitude of the output voltage. By investigating the −3 dB point in Figure 16, the measurement bandwidth of the magnetometer can be calculated. The bandwidth of the prototype in atmospheric environment is 245 Hz, while the bandwidth in 10 Pa vacuum environment is only 0.26 Hz. Therefore although the magnetometer operated in vacuum environment has high Quality Factor and sensitivity, it can only be used to detect the static magnetic field.

### 5.3. The Resolution and Sensitivity

**Figure 17.**
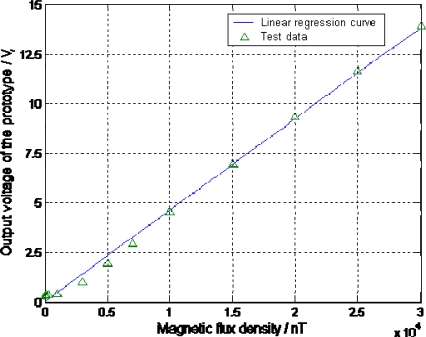
The Output performance of the magnetometer prototype.

To measure the performance of the magnetometer prototype, such as the sensitivity and resolution, we tested the sensor in the Space Magnetic Environment Laboratory. The Helmholtz coil in the laboratory can generate high-precision static magnetic flux density from 0 to 100 μT with 10 nT accuracy and 1 nT resolution. The experiment setup is 10 Pa vacuum environment, 150 mV driving voltage amplitude. The test data is shown in Table 2 when applying the magnetic flux density from 0 μT to 30 μT. Figure 17 shows the linear regression curve of the test data.

The linear regression in the whole measurement range from 0 μT to 30 μT can be expressed as:

(8) y=0.000461x+0.0294

Herein *y* is the output voltage, *x* is the applied magnetic flux density in nT. The linear correlation coefficient is 0.997, which is very close to 1.0. It means the output voltage is proportional to the applied magnetic flux density. In Figure 17, the output voltage is observed non-linear from 0 nT to 3000 nT. The sensitivity for the linear range from 3000 nT to 30000 nT is of 481 mV/μT. Therefore the tested prototype is only appropriate to detect the magnetic flux density between 3000 nT and 30000 nT.

We think the main reason leading to the non-linear response from 0 nT to 3000 nT is due to the vibration of the vacuum pump. Since the magnetometer prototype operates in a resonant condition, such vibration will largely influence the output voltage when the magnetic flux density is small. In addition, the residual magnetic field in the Helmholtz coil, which might be generated by the short-term change of the earth magnetic field, may also decrease the linearity and accuracy. Research on these possible reasons will soon be done in the future. During the experiment, a 10 mV change of the output signal is the minimum voltage change that could be detected. Therefore, according to Table 2, the resolution of the prototype can achieve 30 nT in the linear measurement range between 3000 nT and 30000 nT.

## 6. Conclusions

A torsional resonant magnetometer has been designed and fabricated using conventional MEMS technology and a silicon-to-glass anodic bonding process. The vibration amplitude of the silicon resonator, which is proportional to the value of the magnetic flux-density, is converted into the differential change of sensing capacitances. The low-resistivity silicon resonator directly acts as the capacitance electrode, which largely simplifies the fabrication process. By choosing optimal parameters and taking into account the squeeze film damping effect of the air gap, the sensitivity and resolution of the magnetometer is largely increased. The driving and detection circuit is designed for the magnetometer, which can be integrated with the sensor to fabricate the prototype. According to the performance test of the prototype in a 10 Pa vacuum environment, the Quality factor reaches over 2,500, the sensitivity reaches 481 mV/μT and the resolution can be increased to 30 nT in the linear measurement range from 3,000 nT to 30,000 nT. With such a high sensitivity and resolution, the magnetometer can be applied in the geomagnetic field measurement to assist the attitude control of micro aircrafts. To further improve the magnetometer’s performance, future works could be focused on studying the non-linear response and increasing the sensor’s reliability and consistency.

## Figures and Tables

**Table 1.** Test results of the initial static capacitance (pF).

**TYPE\Number**	**1**	**2**	**3**	**4**
M1	2.7	2.7	2.7	2.7
M2	2.7	2.7	2.6	2.6
M3	2.8	2.7	2.7	2.7
M4	2.7	2.6	2.6	2.6
M5	2.7	2.6	2.7	2.6

**Table 2.** The test data of the magnetometer prototype.

**Magnetic flux density (nT)**	**Output voltage (V)**	**Magnetic flux density (nT)**	**Output voltage (V)**
0	0.33	7000	2.92
30	0.35	10000	4.53
100	0.36	15000	6.97
300	0.37	20000	9.33
1000	0.40	25000	11.64
3000	0.97	30000	13.93
5000	1.93		
